# Massive hydatid cysts in the lung and liver of a 4‐year‐old boy

**DOI:** 10.1002/ccr3.2763

**Published:** 2020-03-09

**Authors:** Uet Yu, Meng Yi, Xiuli Yuan, Feiqiu Wen, Sixi Liu

**Affiliations:** ^1^ Department of Hematology and Oncology Shenzhen Children’s Hospital Shenzhen China

**Keywords:** *Echinococcus granulosa*, hydatid disease, parasite, pediatric

## Abstract

Hydatids may persist for years if left undiagnosed. Early identification is challenging, especially in patients from nonendemic regions. It is crucial for clinicians to be aware of the presence of the disease to avoid devastating outcomes.

## Graphical abstract

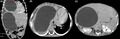

## 1 CASE

Hydatid diseases are not common in children who reside outside the epidemic regions. Asymptomatic infections could persist for years and are often misdiagnosed. A comprehensive review of cyst history and imaging is vital for the early diagnosis of echinococcosis.

**Figure 1:**
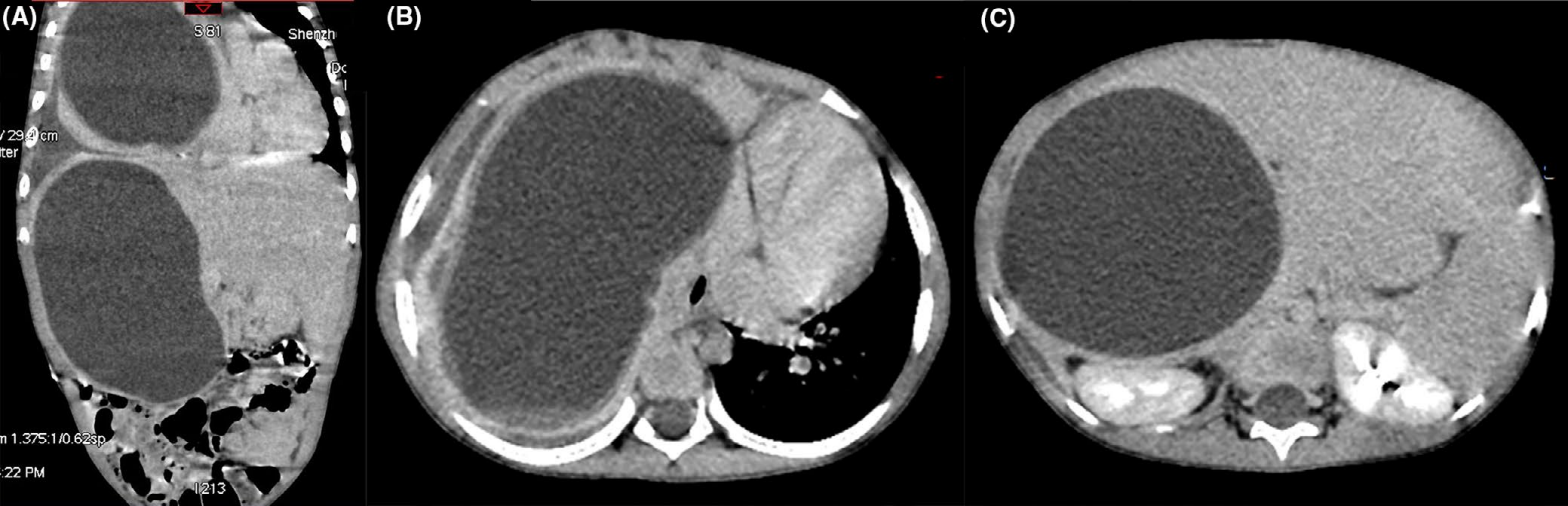
Enhanced computed tomography (CT) scans revealed massive hydatid cysts in the parenchymal portions of the right lung and liver of the patient: A, lung and liver hydatid cysts on the coronal plane, B, lung hydatid cyst on the axial plane, and C, liver hydatid cyst on the axial plane

**Figure 2:**
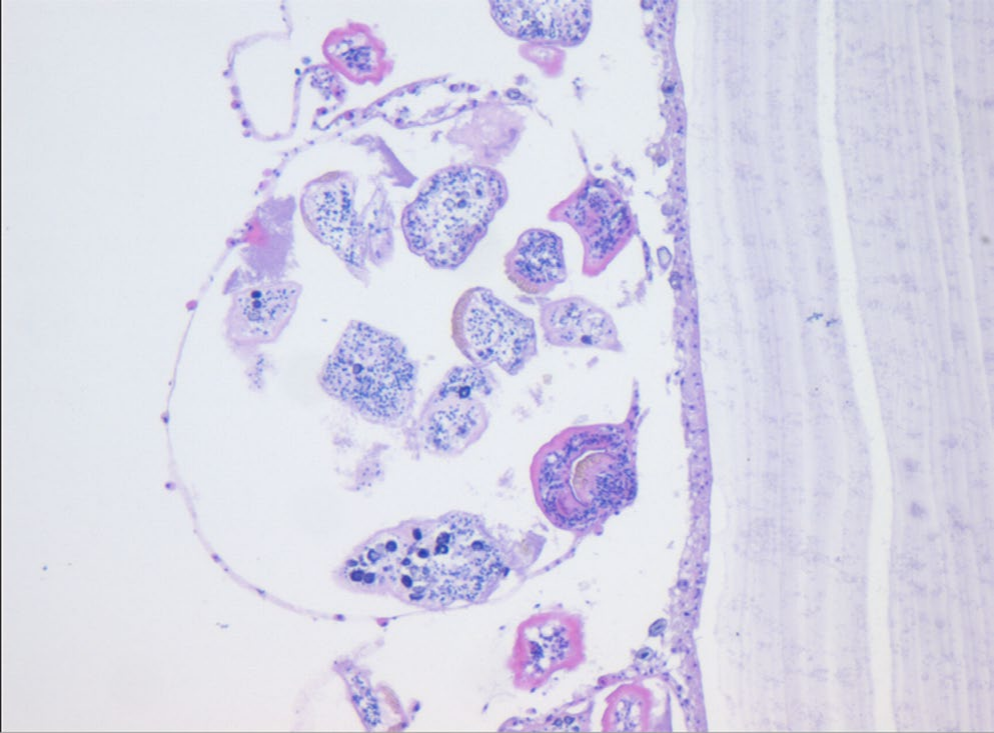
Hematoxylin and eosin (H&E) staining of the hydatid cysts removed from the liver showed *Echinococcus granulosa* protoscolices

A 4‐year‐old boy presented with the gradual enlargement of abdominal masses for more than a year, and persistent fever and tachypnea for a few weeks. He was suspected of neuroblastoma due to elevated levels of serum neuron‐specific enolase. However, he had an elevation of absolute eosinophil count of 0.88 × 10^9^/L, which accounted for 5.6% of the total white blood cells. Chest and abdominal enhanced computed tomography (CT) scans showed large, cystic, lobulated structures in the parenchymal portion of the right lung and liver (Figure 1), suggesting hydatid disease. Antibodies against Echinococcus were positive. Praziquantel and albendazole were administered. All cysts were completely removed by an open echinococcectomy without resection of the residual cavities in the lung and liver. *Echinococcus granulosa* protoscolices were found in the cysts (Figure 2). This patient remained on the anthelmintic treatment with oral albendazole for 6 months after the operation.

Echinococcosis is uncommon in patients with no contact history. We determined that the patient had traveled to Xinjiang Province, which has a high incidence of echinococcosis more than 2 years ago.^1^ This case is expected to alert clinicians to uncommon infections outside the epidemic regions. Delays in treatment may cause devastating outcomes, especially in young children.^2^

## CONFLICT OF INTEREST

The authors had no conflict of interests to declare.

## AUTHOR CONTRIBUTIONS

UY: prepared and wrote this manuscript. MY and XY: were responsible for the clinical consultation of this patient and reviewed this manuscript. FW and SL: took full responsibility of the care of the patient and contributed for the revision of this manuscript.
